# Are decisions using cost-utility analyses robust to choice of SF-36/SF-12 preference-based algorithm?

**DOI:** 10.1186/1477-7525-3-11

**Published:** 2005-03-04

**Authors:** A Simon Pickard, Zhixiao Wang, Surrey M Walton, Todd A Lee

**Affiliations:** 1 Center for Pharmacoeconomic Research, College of Pharmacy, Room 164, 833 S. Wood St (MC886), University of Illinois at Chicago, Chicago, IL, 60612 USA; 2 Midwest Center for Health Services and Policy Research, Hines VA Hospital, Hines, Illinois, USA; 3 Center for Healthcare Studies and Division of General Internal Medicine, Northwestern University Feinberg School of Medicine, Chicago, IL, USA

## Abstract

**Background:**

Cost utility analysis (CUA) using SF-36/SF-12 data has been facilitated by the development of several preference-based algorithms. The purpose of this study was to illustrate how decision-making could be affected by the choice of preference-based algorithms for the SF-36 and SF-12, and provide some guidance on selecting an appropriate algorithm.

**Methods:**

Two sets of data were used: (1) a clinical trial of adult asthma patients; and (2) a longitudinal study of post-stroke patients. Incremental costs were assumed to be $2000 per year over standard treatment, and QALY gains realized over a 1-year period. Ten published algorithms were identified, denoted by first author: Brazier (SF-36), Brazier (SF-12), Shmueli, Fryback, Lundberg, Nichol, Franks (3 algorithms), and Lawrence. Incremental cost-utility ratios (ICURs) for each algorithm, stated in dollars per quality-adjusted life year ($/QALY), were ranked and compared between datasets.

**Results:**

In the asthma patients, estimated ICURs ranged from Lawrence's SF-12 algorithm at $30,769/QALY (95% CI: 26,316 to 36,697) to Brazier's SF-36 algorithm at $63,492/QALY (95% CI: 48,780 to 83,333). ICURs for the stroke cohort varied slightly more dramatically. The MEPS-based algorithm by Franks et al. provided the lowest ICUR at $27,972/QALY (95% CI: 20,942 to 41,667). The Fryback and Shmueli algorithms provided ICURs that were greater than $50,000/QALY and did not have confidence intervals that overlapped with most of the other algorithms. The ICUR-based ranking of algorithms was strongly correlated between the asthma and stroke datasets (r = 0.60).

**Conclusion:**

SF-36/SF-12 preference-based algorithms produced a wide range of ICURs that could potentially lead to different reimbursement decisions. Brazier's SF-36 and SF-12 algorithms have a strong methodological and theoretical basis and tended to generate relatively higher ICUR estimates, considerations that support a preference for these algorithms over the alternatives. The "second-generation" algorithms developed from scores mapped from other indirect preference-based measures tended to generate lower ICURs that would promote greater adoption of new technology. There remains a need for an SF-36/SF-12 preference-based algorithm based on the US general population that has strong theoretical and methodological foundations.

## Background

Health-related quality of life (HRQL) measures have many applications, including the measurement of population health status and outcomes of medical interventions that subsequently can be applied to economic evaluations of health care interventions. One such method of economic evaluation, cost utility analysis (CUA), is a special form of cost effectiveness analysis that evaluates incremental costs and effects of an intervention by assessing health effects using quality-adjusted life years (QALYs) [1]. QALYs incorporate both length of life and quality of life into a single metric, and are calculated by summing the time periods individuals spend in different health states, weighted by the qualities of the health states [2]. Because new therapies are typically more expensive than standard therapies, CUA has gained prominence as a method to inform decision makers who seek to compare the tradeoff in incremental costs and gains in health conferred by new treatment choices within and across disease states.

Optimally, CUA is used to guide the allocation of resources on a societal level. The Panel on Cost Effectiveness in Health and Medicine recommended that community preferences for health states collected from a representative sample of the US general population should be "the most appropriate ones for use in a Reference Case analysis" for US decision makers [2]. Such an approach is facilitated by indirect preference-based generic measures of health-related quality of life (HRQL) such as the Quality of Well-Being Scale [3], Health Utilities Index [4,5], and EQ-5D [6,7], as opposed to elicitation of preferences directly from patients using techniques such as the standard gamble, rating scale, and the time trade-off. Indirect preference-based HRQL measures typically generate index-based single summary scores for health states described by the instrument's classification system using an algorithm based on preferences of the community or general population.

An important development in health services research has been the emergence of algorithms that generate single preference-based summary scores based on items, domain scores, or summary scores from the Short Form 36 (SF-36) [8] and SF-12, a 12-item subset of the SF-36 [9]. While many SF-12 and SF-36 datasets are available due to the widespread use of this family of health assessment measures in clinical trials and population health surveys, their value for application to economic evaluations has been previously limited due to an absence of a scoring algorithm that could generate QALYs from SF-12 and SF-36 response sets. The preference-based algorithms provide an opportunity to use SF-36 and SF-12 data in CUA. As of 2004, 10 published algorithms were identified in the literature that were based on SF-36 or SF-12 items, subscale scores, or summary scores [10-18]. Each preference-based algorithm is unique, derived from different modeling approaches, items/domains, data and/or sources of preferences. Several of these algorithms have been compared in studies, and found to differ from one another and from valuations directly elicited from patients [19-22]. Studies have used some of the algorithms to conduct CUA [23-27], which may be used to inform health care resource allocation. Although the algorithms are known to produce different results, their impact on incremental cost-utility ratios (ICURs) and related decision-making in health care have not been clearly demonstrated.

The purpose of this study was to examine how choice of algorithm for the SF-36/SF-12 might affect decision-making. The specific objectives for the study were to calculate ICURs by applying each algorithm to data from 2 different studies that included longitudinal assessments of the SF-36, to compare the ranking of each algorithm-based ICUR across conditions, and finally to interpret whether differences in ICURs generated by each algorithm had the potential to affect decision making. There were two specific hypotheses. First, ICURs calculated from different algorithms were expected to differ because preferences derived from those algorithms had been found to be different [19]. Second, the rank ordering of ICURs was expected to be similar between the conditions, stroke and asthma, examined in the CUA simulations.

## Methods

### Data sources

To illustrate the outcomes of CUA using the different SF-36 algorithms, data with empiric responses to the SF-36 from patients were used from two different sources and conditions: (1) a clinical trial of adults with asthma [19]; and (2) a longitudinal study of health-related quality of life (HRQL) after stroke [28]. The study of asthma patients was a 12-month randomized controlled trial conducted in inhaled corticosteroid naïve adult patients with mild persistent to moderate persistent asthma that compared two inhaled corticosteroid treatments, triamcinolone acetonide hydrofluoroalkane and fluticasone propionate. Patient included in this trial were ≥ 18 years old, had had a forced expiratory volume in 1 second ≥ 60% of their predicted value after withholding inhaled β-agonists, and had had airway reversibility of ≥ 15% following the administration of an inhaled β-agonist. For the purpose of this analysis, responses to the SF-36 at baseline and 12 months were used.

The second source of data was a natural history of HRQL after stroke. Stroke patients who were hospitalized with a confirmed ischemic stroke and consented to participate were included. Patients were excluded if they were ≤ 18 years old, could not comprehend English-based questionnaire, lived > 150 kilometers from Edmonton, Alberta, had hemorrhagic or lower brain stem stroke, coma, global or Wernicke's aphasia, or life expectancy was less than 6 months for any medical reason. Patients were enrolled in the study within two weeks of stroke and no later than 3 weeks after stroke. Health status measures, including the SF-36, were self-assessed by patients. For this analysis, responses to the SF-36 at baseline and 6 months were used. Both the stroke and asthma studies used version 1 of the SF-36.

### Measures

The SF-36 has been traditionally described as a psychometrically-derived generic health status profile, with 8 subscales and two summary scores, the physical component summary (PCS-36) score and the mental component summary (MCS-36) score. The eight domains include physical functioning (PF), role limitations-physical (RP), bodily pain (BP), general health (GH), mental health (MH), role limitations-emotional (RE), vitality (VT), and social functioning (SF). The SF-12 is a shorter, 12-item version of the SF-36 that does not generate domain scores but provides summary scores, the PCS-12 and MCS-12, that are highly predictive of the PCS-36 and MCS-36 [9]. Scores of the 8 subscales range from 0 to 100. The summary scores (i.e. PCS-36, MCS-36) have a mean score of 50 and a standard deviation of 10. Similarly, the PCS-12 and MCS-12 summary scores have a mean of 50 and a standard deviation of 10.

### Preference-Based Algorithms for SF-36 and SF-12

Nine publications that derived 10 unique preference-based algorithms for the SF-36 or SF-12 were identified (Table 1) [10-18]. Four algorithms were identified that mapped scores for the SF-36, and 6 algorithms mapped scores for the SF-12. The mapping approach was described as 1^st ^generation if the algorithm was derived from directly elicited preferences, and denoted as 2^nd ^generation mapping if the SF-12/SF-36 algorithm was based upon scores from an indirect preference-based HRQL measure, such as the EQ-5D. Note that these algorithms relate to the most recently advocated algorithms, as several authors published earlier algorithms and subsequently published updates (e.g. Shmueli) [16]. For brevity, each published algorithm is identified by the name of the first author.

**Table 1:** Summary of SF-12/SF-36 preference-based algorithms

	Theoretical Range*					
					
Algorithm	Minimum	Maximum	Original source of Preferences	Source of value (country)	Source of sample (country)	Sample Size
Brazier (SF-12)	0.35	1.00	1^st ^generation – SG	UK	UK	836
Lundberg (SF-12)	0.27	0.97	1^st ^generation – VAS	Sweden	Sweden	4,180
Franks (SF-12)	-0.24	0.92	2^nd ^generation – EQ-5D	UK	US	240
Franks (SF-12)	-0.09	0.96	2^nd ^generation – HUI3	Canada	US	240
Franks (SF-12)	-0.07	0.98	2^nd ^generation – EQ-5D	UK	US	12,998
Lawrence (SF-12)	0.15	1.01	2^nd ^generation – EQ-5D	UK	US	14,580
Shmueli (SF-36)	0.23	1.00	1^st ^generation – VAS	Israel	Israel	2,505
Brazier (SF-36)	0.30	1.00	1^st ^generation – SG	UK	UK	836
Fryback (SF-36)	0.59	0.84	2^nd ^generation – QWB	US	US	1,356
Nichol (SF-36)	0.24	1.05	2^nd ^generation – HUI2	Canada	US	6,921

*Maximum and minimum scores are based on best and worst responses to all items on the SF-36 and SF-12. For the Lundberg algorithm, minimum obtained is based on male, ≥ 80 years of age, while maximum is based on female, <30 years of age. For the Nichol algorithm, the minimum is based on 100 years of age, while maximum is based on 0 years of age.

Brazier and colleagues constructed an econometric model for predicting health state valuations by first revising the SF-36 into a health status measure with 6 domains called the SF-6D [10]. Using a variant on the standard gamble, 249 health states defined by the SF-6D were valued by a representative sample of the UK general population. Ordinary least squares (OLS) models were estimated to predict all 18,000 SF-6D health states. The Brazier (SF-36) algorithm used for the present study was based on the parsimonious consistent model, the preferred specification for model 10. The same data and a similar approach was used to estimate an algorithm based on the SF-12 [17].

Fryback and colleagues predicted Quality of Well-being Index (QWB) scores from SF-36 domain scores using data collected from the Beaver Dam Health Outcomes Study [12]. A six-variable regression model with three main effects (PF, MH, and BP) and three interaction terms (GH*RP, PF*BP, and MH*BP) is used to estimate preferences.

Nichol and colleagues mapped the SF-36 to the preference-based Health Utility Index Mark 2 (HUI2). They estimated HUI2 scores from SF-36 domain scores and sociodemographic variables from a sample of Southern California Kaiser Permanente members [15]. The Nichol method used OLS models, retaining statistically significant parameter estimates that included all eight domains of the SF-36 and age of the respondent.

Shmueli updated an examination of the relationship between Visual Analog Scale (VAS) ratings and SF-36 domains provided in a population health survey in Israel by predicting VAS values from SF-36 domains using linear and non-linear regression models [16]. The model was anchored such that scores of 100 on all 8 SF-36 domains would result in a VAS score of 100. The present study used the anchored algorithm that included statistically significant coefficients for PF, MH, VT, and GH.

Franks and colleagues mapped the SF-12 to the EQ-5D Index and HUI3 using a convenience sample of 240 low-income, predominantly Latino and black patients visiting a community health center in New York [11]. Two equations were separately developed that mapped the PCS-12 and MCS-12 onto EQ-5D and HUI3 scores using OLS models. Described as a pilot, the authors observed that the level of explained variance was consistent with the Fryback and Nichol studies (between 50–60%). Franks led a second investigation, again mapping the SF-12 to EQ-5D scores, using data from the Medical Expenditure Panel Survey (MEPS) [18]. The algorithm based upon SF-12 responses that did not include demographic variables was utilized for the present study. In a similar analysis, Lawrence and colleagues predicted the EQ-5D scores from the SF-12 using MEPS data [13]. A series of 2-variable, 3-variable, and 6-variable models, based on functional variations on, and interactions between, the PCS-12 and MCS-12 were developed. The 2-variable model was advocated for its simplicity and predictive ability across a diverse set of subgroups in the validation set.

Finally, Lundberg and colleagues investigated the relationship of preference-based measures and the SF-12 based on self-assessed HRQL from a random sample of residents in Uppsala County of Sweden [14]. Linear regression models were used to predict valuations from 11 of the 12 items on the SF-12 (excluding the global health item), age, and gender. When using proportion explained variance as a criterion, the reduced VAS-based model that retained only significant coefficients was recommended, with 50% of variance explained by the model.

### Data analysis

Empiric data for stroke and asthma were used to ensure that actual health state changes were represented. The present analysis was based on patients who completed both pre- and post- assessments and had no missing items. After the scoring algorithms were applied to SF-36 responses using the 10 algorithms [10-18], the change in utility was transformed into QALYs, with the assumption that the incremental gain/lose in health state utility was realized for a 1-year period. QALYs were calculated using the area under the curve (AUC) approach. We assumed incremental costs associated with the intervention were $2000 per year greater than standard treatment in both the stroke and asthma patients. Such costs over standard treatment were considered reasonable approximations for the costs of an innovative treatment in asthma and stroke, and although distributions of costs could have been used to further simulate a "realistic CUA", but would further complicate the paper without contributing to the main purpose of this study. The incremental cost utility ratio (ICUR) between the intervention and control groups was calculated by dividing incremental costs by gain in QALYs. The algorithms were ranked based on ICURs for each condition.

The pre/post domain and preference-based scores were described for both study groups visit using means and standard deviations. The 95% confidence intervals (CIs) for ICURs were based on the CIs for the preference scores. The pre/post change scores were evaluated with paired *t*-tests. The rank order of the ICURs was compared between the asthma and stroke groups using Spearman's correlation coefficient (r_s_). P-values < 0.05 were considered statistically significant.

## Results

Of the 304 patients enrolled in the asthma study, 220 (72.4%) completed both the baseline and final SF-36 assessment. The stroke study had 81 of 124 initial respondents (65.3%) complete the SF-36 at baseline and final follow-up. In comparison to the patients in the asthma study, patients in the stroke study were older (mean age 67.4 years vs. 39.1 years) and had much lower mean average PF, RP, SF, and PCS scores (Table 2). Positive change was observed on all 8 domains of the SF-36 in the asthma patients from the baseline to the end of the study (all p-values < 0.01). Stroke patients showed trend towards improvement on all 8 domains, with significant improvement on all domains (p-values < 0.01) with the exception of GH and BP (p-values > 0.05).

**Table 2:** Demographics Characteristics and SF-36 Scores

	Asthma Patients (n = 220)				Stroke Patients (n = 81)			
	Baseline Assessment		Final Assessment		Baseline Assessment		Final Assessment	
	
	Mean	(SD)	Mean	(SD)	Mean	(SD)	Mean	(SD)
Age	39.1	(12.6)			67.4	(14.4)		
Female (%)	55				49			
GH	59.4	(18.8)	69.4^‡^	(19.0)	54.4	(18.4)	56.8	(22.2)
BP	66.4	(23.2)	75.5^‡^	(21.8)	62.3	(27.4)	68.8	(30.8)
PF	63.1	(21.9)	81.3^‡^	(21.4)	17.8	(25.9)	41.6^‡^	(33.0)
RE	63.3	(41.4)	79.6^‡^	(34.5)	47.3	(44.7)	68.3^†^	(44.1)
RP	38.1	(40.0)	73.3^‡^	(37.4)	8.3	(23.7)	32.1^‡^	(40.2)
MH	71.2	(17.9)	75.9^‡^	(16.6)	67.2	(19.2)	77.9^‡^	(17.2)
SF	72.6	(22.0)	83.1^‡^	(19.8)	42.7	(26.4)	60.8^‡^	(31.8)
VT	48.8	(20.7)	60.0^‡^	(21.6)	41.5	(17.8)	50.5^†^	(22.8)
PCS	40.1	(9.0)	48.2^‡^	(9.1)	28.9	(8.52)	34.5^‡^	(12.8)
MCS	48.1	(11.1)	50.5^†^	(10.3)	46.4	(11.2)	51.7^†^	(10.8)

^†^p-value < 0.01; ^‡^p-value < 0.001, based on *t*-test for dependent samples

According to the preference-based summary scores, all patients in both studies demonstrated statistically significant improvement from baseline to the end of the study (p-value < 0.001) (Table 3). In the asthma study, the mean (SD) change in preference scores ranged from 0.063 (0.117) to 0.130 (0.159). In the stroke study, change scores ranged between 0.055 (0.124) and 0.143 (0.215).

**Table 3:** Preference-Based Scores for Asthma and Stroke Samples using SF-36 Algorithms

	Baseline Assessment (T_i_)		Final Assessment (T_f_)		Difference (T_f_-T_i_)		95% CI	
**Asthma (n = 220)**	Mean	(SD)	Mean	(SD)	Mean	(SD)	Lower	Upper
Brazier (SF-36, SG)	0.694	(0.101)	0.757	(0.113)	0.063^‡^	(0.117)	0.048	0.082
Brazier (SF-12, SG)	0.724	(0.116)	0.789	(0.119)	0.065^‡^	(0.125)	0.047	0.078
Fryback (SF-36, QWB)	0.655	(0.063)	0.721	(0.072)	0.066^‡^	(0.070)	0.057	0.075
Nichol (SF-36, HUI2)	0.765	(0.123)	0.840	(0.118)	0.075^‡^	(0.114)	0.060	0.090
Shmueli (SF-36, VAS)	0.683	(0.124)	0.766	(0.130)	0.084^‡^	(0.111)	0.069	0.098
Lundberg (SF-12, VAS)	0.667	(0.113)	0.759	(0.119)	0.091^‡^	(0.117)	0.076	0.107
Franks (SF-12, EQ-5D)	0.699	(0.181)	0.814	(0.152)	0.115^‡^	(0.169)	0.093	0.138
Franks (SF-12, HUI3)	0.643	(0.170)	0.764	(0.173)	0.121^‡^	(0.176)	0.098	0.144
Franks (SF-12, EQ-5D, MEPS)	0.667	(0.174)	0.797	(0.163)	0.129^‡^	(0.167)	0.107	0.151
Lawrence (SF-12, EQ-5D)	0.667	(0.158)	0.798	(0.159)	0.130^‡^	(0.159)	0.109	0.152
**Stroke (n = 81)**								
Shmueli (SF-36, VAS)	0.602	(0.115)	0.656	(0.155)	0.055^‡^	(0.124)	0.027	0.082
Fryback (SF-36, QWB)	0.548	(0.060)	0.616	(0.100)	0.069^‡^	(0.094)	0.048	0.089
Lundberg (SF-12, VAS)	0.512	(0.108)	0.592	(0.155)	0.080^‡^	(0.156)	0.045	0.114
Brazier (SF-12, SG)	0.609	(0.099)	0.696	(0.145)	0.087^‡^	(0.152)	0.054	0.121
Nichol (SF-36, HUI2)	0.656	(0.110)	0.745	(0.147)	0.089^‡^	(0.143)	0.058	0.121
Brazier (SF-36, SG)	0.552	(0.087)	0.669	(0.139)	0.116^‡^	(0.137)	0.086	0.147
Franks (SF-12, HUI3)	0.482	(0.150)	0.615	(0.200)	0.133^‡^	(0.200)	0.089	0.177
Lawrence (SF-12, EQ-5D)	0.491	(0.132)	0.626	(0.204)	0.134^‡^	(0.194)	0.091	0.177
Franks (SF-12, EQ-5D)	0.478	(0.199)	0.618	(0.232)	0.139^‡^	(0.233)	0.088	0.191
Franks (SF-12, EQ-5D, MEPS)	0.472	(0.165)	0.615	(0.219)	0.143^‡^	(0.215)	0.096	0.191

^‡^p-value < 0.001, based on *t*-test for dependent samples

NB: algorithms are ordered from smallest to largest difference score for each condition

Table 4 shows the results from the two sets of CUA simulations, and the rank order of the algorithms. As the incremental cost of $2000 is held constant across the algorithms, the differences in QALYs are mirrored by the differences in ICURs. In the asthma patients, estimated ICURs ranged from Lawrence's SF-12 algorithm  at $30,769/QALY (95% CI: 26,316 to 36,697) to Brazier's SF-36 algorithm at  $63,492 (95% CI: 48,780 to 83,333). ICURs for the stroke cohort varied slightly more dramatically. The MEPS-based algorithm by Franks et al. provided the lowest ICUR at $27,972/QALY (95% CI: 20,942 to 41,667). The Fryback and Shmueli algorithms provided ICURs that were greater $50,000/QALY and did not have confidence intervals that overlapped with most of the other algorithms. The rank order of algorithms based on ICUR was similar across the two conditions, with r_s _= 0.60 (p-value < 0.10).

**Table 4:** Ranking of SF-36/SF-12 Algorithm by Estimated Incremental Cost Utility Ratio

	Incremental Cost	1 year QALYs Gained	ICUR ($/QALY) [95% CI]	Rank
**Asthma**				
Lawrence (SF-12, EQ-5D)	$2000	0.065	30 769 [26 316, 36 697]	1
Franks (SF-12, EQ-5D, MEPS)	$2000	0.065	31 008 [26 490, 37 383]	2
Franks (SF-12, HUI3)	$2000	0.061	33 058 [27 778, 40 816]	3
Franks (SF-12, EQ-5D)	$2000	0.058	34 783 [28 986, 43 011]	4
Lundberg (SF-12, VAS)	$2000	0.046	43 956 [37 383, 52 632]	5
Shmueli (SF-36, VAS)	$2000	0.042	47 619 [40 816, 57 971]	6
Nichol (SF-36, HUI2)	$2000	0.038	53 333 [44 444, 66 667]	7
Fryback (SF-36, QWB)	$2000	0.033	60 606 [53 333, 70 175]	8
Brazier (SF-12, SG)	$2000	0.033	61 538 [51 282, 85 106]	9
Brazier (SF-36, SG)	$2000	0.032	63 492 [48 780, 83 333]	10
**Stroke**				
Lawrence (SF-12, EQ-5D)	$2000	0.067	29 851 [22 599, 43 956]	3
Franks (SF-12, EQ-5D, MEPS)	$2000	0.072	27 972 [20 942, 41 667]	1
Franks (SF-12, HUI3)	$2000	0.067	30 075 [22 599, 44 944]	4
Franks (SF-12, EQ-5D)	$2000	0.070	28 777 [20 942, 45 455]	2
Lundberg (SF-12, VAS)	$2000	0.040	50 000 [35 088, 88 889]	8
Shmueli (SF-36, VAS)	$2000	0.028	72 727 [48 780, 148 148]	10
Nichol (SF-36, HUI2)	$2000	0.045	44 944 [33 058, 68 966]	6
Fryback (SF-36, QWB)	$2000	0.035	57 971 [44 944, 83 333]	9
Brazier (SF-12, SG)	$2000	0.044	45 977 [33 058, 74 074]	7
Brazier (SF-36, SG)	$2000	0.058	34 483 [27 211, 46 512]	5

NB: algorithms are ordered from lowest to highest ICUR in the asthma patients

## Discussion

The development of preference-based algorithms for the SF-36 and SF-12 to facilitate CUA has fostered studies that recognized these preference-based scores can differ from each other and from directly elicited valuations in patients with asthma, hypertension, lung transplantation, and osteoporosis [19,20,29,30]. However, the extent to which the differences might lead to different decisions on implementing or reimbursing for a new technology has been unclear. Using actual health states self-assessed by patients and imputing what might be considered conservative costs for an innovative treatment, our analysis demonstrated that ICURs based on the derivation algorithms can vary dramatically. The 10 algorithms produced a wide range of ICURs that varied more than 2-fold in magnitude for the asthma cohort and almost 3-fold in the stroke study.

Although guidelines or thresholds for decision making based on cost per QALY are contentious, cost-effectiveness thresholds that health care decision makers are willing to accept in health care reimbursement decisions exist, if not explicitly, then implicitly. Some guidance has been published. The National Institute of Clinical Effectiveness (NICE) in the UK has indicated they do not have an explicit threshold [31], while a threshold of around £20,000 to £30,000 per QALY gained (about $37,000 to $55,000 in 2004 US dollars) [32,33] or slightly higher [34] has been cited as the value used in making decisions. Laupacis et al (1992) suggested that a treatment costing less than $20,000/QALY could be considered very cost-effective, a treatment costing between $20,000/QALY and $100,000/QALY was judged acceptable, while a treatment costing more than $100,000/QALY was deemed not likely to be cost-effective [35]. Other studies have suggested that $50,000/QALY provides a threshold for judging cost effectiveness [36,37]. Although arbitrary criteria, the application of any of the cited guidelines to the CUAs illustrated in the present study convey that the choice of algorithm can dictate whether the intervention is considered cost-effective or unacceptable. The choice of algorithm could determine whether a drug is considered for formulary listing, particularly if an emphasis is placed on cost-effectiveness as a criterion by the decision-making committee, as is often done by publicly funded health care systems.

The CUA simulations illustrated how selection of a specific algorithm could lead to a different interpretation of the cost-effectiveness of an intervention. In the asthma cohort, algorithms by Lawrence [13], and the three equations by Franks [11,18] generated relatively smaller ICURs close to a level that may be considered very cost-effective, i.e. $20,000, with 95% confidence intervals that did not bound the $50,000/QALY threshold. In contrast, the Nichol, Fryback, and both Brazier methods produced ICUR point estimates above $50,000/QALYs that would be unacceptable by most guidelines. In stroke, the Lawrence and Franks methods again generated ICURs that would indicate the technology of interest was relatively cost-effective, at $30,000/QALY or less, while the algorithms by Shmueli [16] and Fryback [12] produced ICURs over $50,000/QALY. In examining the robustness of the results, all algorithms produced ICURs below $20,000/QALY when incremental costs for the hypothetical intervention are reduced to less than $500, but algorithm selection becomes critical as incremental costs increase and thresholds such as $20,000/QALYs or $50,000/QALY are crossed.

Changes in the rank order of algorithms between conditions can be explained, not only by differences in the preference-based weighting assigned to each of the domain/summary scores, but several additional factors. There are differences in the SF-36 items or subscales retained by some of the methods. For instance, Brazier's SF-6D based on the SF-36 does not include GH, while the score generated by the Shmueli algorithm is largely influenced by the GH domain. The responsiveness/sensitivity of algorithms appears to be somewhat related to the scale range. It was not surprising that Fryback's method produced relatively larger ICURs that implied the intervention was less cost effective, as Fryback's method had a much smaller range of scale relative to the other algorithms (Table 1). Algorithms that incorporate demographic characteristics, such as the Nichol and Lundberg methods, provide estimates that are influenced by age of the cohort and could contribute to changes in their rank order.

In order to provide some guidance in the selection of preference-based algorithms for the SF-36 and SF-12 the algorithms were appraised in the context of their theoretical and methodological foundations, source of community-based preferences, and their relative potential to enhance or deter the uptake of new technology. The study results clearly illustrated that choice of algorithm can affect the estimated ICUR, and that there was a tendency for the Fryback and Shmueli methods to generate higher estimates of ICURs relative to the other algorithms. From a third-party payer perspective, algorithms generating higher ICURs would appeal to third-party reimbursement decision makers with short-term budget constraints, as algorithms that generate higher ICURs provide less encouraging evidence in the adoption of new technology when considered in the context of the $/QALY benchmarks previously discussed. The Panel on Cost Effectiveness of Health and Medicine [2] recommended that preference-based measures have a theoretical basis and represent community-based preferences. The Brazier algorithms are arguably most favorable on a theoretical basis. Only the Brazier, Lundberg, and Shmueli algorithms were based on preferences directly elicited from the general populace, i.e. first generation. The Lawrence, Franks, Nichol, and Fryback methods mapped the SF-12/SF-36 onto scores obtained from indirect utility-based measures, e.g. EQ-5D, HUI2, to derive what we termed "second generation" preference-based algorithms. Such an approach is limited by differences in the descriptive systems [17]. Interestingly, algorithms derived from directly elicited valuations of health states (i.e. first-generation mapping) tended to generate smaller magnitudes of change compared to the algorithms that mapped the SF-36/12 using other indirect utility measures (i.e. second-generation mapping). One explanation for the second-generation algorithms producing larger change scores is that several of them were derived from the utility scores of the HUI3 and EQ-5D, which have broader scale ranges compared to the SF-6D [38].

A further consideration is the theoretical foundation for the elicitation technique used in the valuation study. Only Brazier employed methodology using the SG technique for first-generation mapping. The SG has the most appeal in economic theory due to its foundations in Expected Utility Theory (EUT), although it has been suggested that the axioms of EUT are empirically flawed [39], and requires the respondent possess a rudimentary understanding of probabilities. Scores generated by Lundberg, Shmueli, and Fryback methods were based on first or second generation mapping of the SF-12/SF-36 onto scores from rating scales. Rating scales have been criticized for lack of theoretical basis in economics [39,40], as a rating scale is not a choice-based technique and its ability to represent preferences on a cardinal scale is debatable. In contrast, the TTO and SG are choice-based techniques that generate utilities [2]. Lundberg utilized a variant of the TTO in addition to the VAS, but the complexity of the TTO task does not lend itself to a mail survey design. Lundberg observed that the TTO models did not perform as well as the VAS. Most of the algorithms were developed using self-assessed preferences for health status from a general population where severe states are rare, rather increasing the representation of more severe health states by statistical design, as done by Brazier [10,17] and by other developers of preference-based measures [4,7].

Given that HRQL may be valued differently between countries [41], an algorithm based on the preferences of a representative sample of the general population for the country of interest would be most desirable for resource allocation decision-making on the societal level, e.g. when the payer is the national ministry of health. The algorithms for the SF-36 find their preference-based origins from a diverse range of national sources. The algorithm by Shmueli was based on valuations obtained from representative samples of the Israeli Jewish population [16], while Lundberg's algorithm was based on valuations from the Sweden populace [14]. Brazier's utilities for health states were elicited from respondents in the United Kingdom [10]. The preferences for algorithms derived by Franks (EQ-5D) [11] and Lawrence [13] were mapped from the EQ-5D scoring function derived from the general population in the United Kingdom [7]. Fryback [12] mapped scores from the QWB that were based on community-based preferences from San Diego, California, USA [3]. The Nichol [11,15] and Franks [11] algorithms were mapped from the utility-based scores of the HUI2 and HUI3 systems, respectively, that were originally elicited from respondents in Ontario, Canada [4,5]. Nichol and Lundberg algorithms may not be considered as representing general population values because they include demographic variables such as age and/or gender. At present, only the Fryback algorithm has preferences originating from a community in the US, albeit a second generation mapping of those preferences.

Among the algorithms presently available, Brazier's algorithms for the SF-12 and SF-36 appear to be most favorable because of their methodological and theoretical basis. From the perspective of the third party reimbursement decision maker, the Brazier algorithms are not among those that tend to encourage adoption of new technology, tending to provide relatively higher estimates of ICUR. For those decision-makers, those algorithms would appear to be more fiscally conservative in the sense that they would not promote the adoption new technology any more than the other methods according to the results of this study. Although similar estimates are obtained using the Brazier SF-36 and SF-12 algorithms [17], it would be preferable to utilize the Brazier SF-36 algorithm rather than the SF-12-based algorithm if responses to the SF-36 are available because of the richer information afforded by the descriptive system. If alternative algorithms are used for CUA, it may be suggested to test robustness of conclusions by sensitivity analysis using Brazier's SF-36 or SF-12-based algorithms. At present, no SF-36/SF-12 algorithm has been published based on the first generation preferences of the US general population. As there is evidence to support health states valuations by the general US population differ from other countries[41], this represents an opportunity for future research leading to the development of an algorithm specific to the US as well as for other countries.

Note that there are several limitations and assumptions to the CUAs simulated in this paper. The primary purpose was to determine whether choice of preference-based algorithm applied to SF-36/SF-12 data has the potential to change the conclusion of a CUA; hence, aspects of the CUA not central to the purpose were simplified. For example, incremental costs were assumed to be constant, whereas in reality, considerable cost variance would be observed across patients. CUAs were performed in two patient populations, i.e., asthma patients and stroke patients, rather than using a single data set, to enhance the generalizability of the results. The rank order of the algorithms is limited to the datasets examined in this study, however, and comparisons of the algorithms across more diseases/conditions and persons with different demographic characteristics may provide stronger evidence of the rank order "stability". Change scores and thus ICUR estimates depend on baseline health status and the impact of an intervention on the various domains of health as captured by changes in responses to the SF-12/SF-36 items. Note that baseline domain scores for asthma and stroke cohorts were lower than US-based population norms. Several algorithm developers provide caveats for the application of their algorithm, including concerns about profiles severely limited by ceiling or floor effects [12], and inconsistent estimates and overprediction of poorest health states [10]. For instance, the descriptive system of the SF-6D is more concentrated at the milder end of health problems relative to the EQ-5D [42]. These concerns may be particularly relevant to the stroke cohort, where floor effects were observed.

## Conclusion

In summary, SF-36/SF-12 preference-based algorithms tend to generate a wide range of ICURs that can potentially lead to different reimbursement decisions. Brazier's algorithms for the SF-36 and SF-12 had an arguably stronger methodological and theoretical basis, and tended to produce higher ICURs. For decision-makers who consider cost-effectiveness in the decision to reimburse for a new medical technology, selection of an algorithm that generates relatively higher ICURs would provide less convincing evidence of the cost-effectiveness of a new technology and consequently, its uptake. The "second-generation" algorithms mapped from other indirect preference-based measures tended to produce lower ICURs. When an alternative algorithm is selected, sensitivity analysis is recommended using the Brazier SF-12/SF-36 algorithm in order to examine the robustness of CUA. There remains a need for an SF-36/SF-12 algorithm developed from U.S.-based general population preferences with strong methodological and theoretical foundations.

## Authors' contributions

Drs. Pickard and Lee were responsible for the conception of the study and acquisition of data. Mr. Wang analyzed the data. Dr. Pickard and Mr. Wang were involved in the drafting of the article. All authors contributed to the interpretation of results and revising the article for important intellectual content.
